# Characterization of oseltamivir-resistant A(H5N1) clade 2.3.4.4b, genotype D1.1 variants identified in poultry farms of British Columbia, Canada

**DOI:** 10.1080/22221751.2026.2686474

**Published:** 2026-07-08

**Authors:** Maxime Cochin, Yacine Abed, Robert Vendramelli, Katrina Dionne, Catherine Bourassa, Geneviève Laroche, Rose Chan, Thang Truong, Anthony Signore, Yohannes Berhane, Marceline Côté, Andres Finzi, Darwyn Kobasa, Guy Boivin

**Affiliations:** a Faculty of Medecine, Université Laval, Québec, Canada; b Research Center in Infectious Diseases, CHU de Québec Research Center-Université Laval, Québec, Canada; c National Microbiology Laboratory, Public Health Agency of Canada, Winnipeg, Canada; d Département de Microbiologie, Infectiologie et Immunologie, Université de Montréal, Québec, Canada; e Centre de Recherche du CHUM, Université de Montréal, Québec, Canada; f Department of Biochemistry, Microbiology, and Immunology, University of Ottawa, Ottawa, Canada; g National Center for Foreign Animal Disease, Canadian Food Inspection Agency, Winnipeg, Canada; h Department of Veterinary Pathology, Western College of Veterinary Medicine, University of Saskatchewan, Saskatoon, Canada; i Department of Animal Science, University of Manitoba, Winnipeg, Canada; j Department of Pathobiology, University of Guelph, Guelph, Canada; k Department of Medical Microbiology and Infectious Disease, University of Manitoba, Winnipeg, Canada

**Keywords:** Avian Influenza, A(H5N1), H5 clade 2.3.4.4b, oseltamivir resistance, NA H275Y

## Abstract

Highly pathogenic avian influenza A(H5N1) viruses of clade 2.3.4.4b, genotype D1.1, are responsible for widespread outbreaks in poultry and continue to cause sporadic, sometimes severe, human infections. Herein, we characterized a wild-type (WT) influenza A(H5N1) D1.1 isolate (BC-H5N1-WT) and its H275Y neuraminidase (NA) variant (BC-H5N1-H275Y), both of which emerged on farms in British Columbia, Canada, during the fall 2024 outbreak. *In vitro* analysis assessed replication kinetics in MDCK cells, with supernatants collected at different days post-infection (p.i.) and titrated by TCID_50_ and qRT-PCR. Neuraminidase inhibitor (NAI) susceptibility was determined by NA inhibition assays, whereas susceptibility to baloxavir acid (BXA) was evaluated by plaque reduction assay. *In vivo* virulence was evaluated in BALB/c mice infected with serial 10-fold dilutions of each virus to monitor weight loss and mortality. Viral titers in lungs, brain, nose, kidney, spleen, and heart were quantified at day 4 p.i. The BC-H5N1-WT virus was susceptible to the four antivirals tested, whereas BC-H5N1-H275Y displayed resistance to oseltamivir and peramivir but remained susceptible to zanamivir and BXA. The BC-H5N1-WT exhibited significantly higher viral replication titers than BC-H5N1-H275Y at all tested time points and showed larger plaque sizes. In mice, BC-H5N1-WT was more virulent with LD_50_ values of 1.78 × 10^3^ PFUs compared to 8.71 × 10^4^ PFUs for BC-H5N1-H275Y, and produced higher viral titers in lungs and other organs. Despite the reduced fitness of the resistant H5N1 D1.1 variant, its emergence in the absence of viral selection pressure underscores the need for continued surveillance.

## Introduction

In late 2021, a novel highly pathogenic avian influenza virus (HPAIV) of H5N1 clade 2.3.4.4b was detected in Newfoundland, Canada, marking its first incursion into North America. This event resulted in a significant impact on wildlife and poultry health and led to major economic losses due to mortality, culling, and trade disruptions in the poultry sector [1]. This variant surprised the scientific community with its extensive spillovers into wild birds and novel mammalian hosts. The clade 2.3.4.4b H5N1 viruses can be further subdivided into distinct genotypes. Most current H5N1-related infections in dairy cows involve variants of B3.13 genotype, whereas outbreaks in wild birds and poultry are currently mainly caused by variants of D1.1 genotype [2]. Human cases with both genotypes have been reported, primarily among healthy farm workers who experienced mild, self-resolving disease such as conjunctivitis [3,4]. Conversely, severe and even fatal cases have also been reported. In particular, H5N1 viruses of D1.1 genotype were associated with severe cases requiring hospitalization [5]. These include a fatal case of a 65-year-old man due to exposure to sick and dead chickens and wild birds in Louisiana [5]. Another severe but non-fatal case involved a 13-year-old Canadian teenager who was admitted to the pediatric intensive care unit at British Columbia (BC) Children’s Hospital with respiratory failure, pneumonia, acute kidney injury, thrombocytopenia, and leukopenia [6]. So far, no human-to-human transmission has been recorded. Nevertheless, the high circulation of 2.3.4.4b A(H5N1) viruses, their adaptability to various animal hosts including domestic mammals and increased human interactions heighten the risk of viral reassortment and host adaptations. These events are a reminder that influenza A(H5N1) viruses remain a potential pandemic threat.

In October 2024, several chicken farms in the province of BC, Canada, faced an outbreak of HPAIV. Whole genome sequencing confirmed that the virus was an H5N1 virus of the clade 2.3.4.4b [7]. Phylogenetic analysis of the hemagglutinin (HA) and neuraminidase (NA) genes confirmed that the viruses involved were reassortant H5 viruses of clade 2.3.4.4b with an avian NA of N1 subtype from a North American wild bird lineage [7]. Notably, this NA has a longer stalk compared to the truncated NA stalk of traditional HPAI H5N1 strains [8]. Fully Eurasian 2.3.4.4b H5N1 viruses also possess this untruncated (long-stalk) NA, differing from the short-stalk NA common in most pre-2020 highly pathogenic H5N1 strains. Interestingly, the NA gene from 8 of the 45 sampled poultry farms harboured the well-described H275Y (N1 numbering) substitution that confers high levels of resistance to oseltamivir (OSV) and peramivir (PER), which are influenza NA inhibitors (NAI). Of note, there was no evidence of OSV or PER use in the related farms that could explain the presence of such H275Y mutants in these animals. Although old H1N1 viruses with the H275Y substitution were associated with a fitness loss [9–11], more recent seasonal and pandemic H1N1 mutant viruses were more fit than the wild-type (WT) virus due to the presence of permissive NA mutations [12–14].

In the present work, we aimed to characterize two influenza A(H5N1) isolates that emerged in the same farm during the recent BC outbreak in 2024: one harbouring the WT NA gene (BC-WT) and the other carrying the NA-H275Y substitution (BC-H275Y). This characterization included genomic analysis, evaluation of NA activity, determination of susceptibilities to several NAIs and to the cap-dependent endonuclease inhibitor baloxavir acid (BXA) as well as evaluation of replicative capacities *in vitro* and in mice.

## Materials and methods

### Cells and viruses

Madin-Darby canine kidney (MDCK) cells were maintained in minimum essential medium (MEM) supplemented with 10% FBS, HEPES and antibiotics. Human embryonic kidney 293T (HEK293T) cells (ATCC) were maintained in Dulbecco’s modified Eagle’s medium (DMEM) (Invitrogen, Carlsbad, CA), supplemented with 10% fetal bovine serum (Invitrogen, Carlsbad, CA).

Avian H5N1 viral strains A/CK/BC/FAV-0266-2/2024 (EPI_ISL_20295037) (BC-WT) and A/CK/BC/FAV-0284-1/2024 (EPI_ISL_19555245) (BC-H275Y) were obtained from the Canadian Food Inspection Agency.

### Phylogenetic analysis and frequencies of NAI resistance mutations

A total of 69,942 complete N1 gene sequences identified in North America between 2000-01-01 and 2026-03-31 were downloaded from the Global Initiative on Sharing All Influenza Data (GISAID) [15]. These include sequences from human A(H1N1)pdm09 strains (n = 50,052), Eurasian origin N1 clade 2.3.4.4b viruses (n = 14,452), North American origin N1 clade 2.3.4.4b viruses (n = 3,965), and North American low pathogenicity avian influenza (LPAI) viruses (n = 1,473). To assess the relative divergence of human A(H1N1)pdm09 and avian N1 lineages, the complete sequence dataset was subset based on translated amino acid identity. After sequences sharing identical amino acid translations were subset to a single sequence, the remaining 14,995 sequences were aligned as above and used to build a maximum-likelihood phylogenetic tree with IQ-TREE v2.4.0 [16]. The tree was estimated with the best fitting model of nucleotide substitution (as determined by ModelFinder, GTR + F + I + R8) and node support for the resulting tree topology was assessed by 5000 ultrafast bootstrap replicates [17,18].

To determine frequencies of amino acid substitutions that confer reduced susceptibility to NAIs, the 69,942 sequences were aligned using MAFFT v7.526 [19], trimmed of the regions flanking the open reading frames, and used to build a phylogenetic tree with Fasttree v2.2.0 using default settings [20]. Amino acid substitutions that have been laboratory tested to confer reduced susceptibility to NAIs in N1 subtypes were identified using FluMut v0.6.4 [21]. The frequency of each substitution was calculated by dividing the number of sequences within a lineage that harbour a given mutation by the total number of sequences for that lineage.

### Generation of 7:1 reassortant influenza viruses

A reverse genetics system using pLLB-A and pLLB-G plasmids was previously developed for the influenza A/Québec/144147/09 pH1N1 virus [22]. The pLLB-A plasmids containing the NA gene of the WT H5N1 isolate or the related H275Y mutant were prepared by RT–PCR amplification and cloning. The resulting recombinant plasmids were sequenced to ensure the absence of undesired mutations by using Sanger sequencing and the ABI 3730 DNA Analyzer. The eight bidirectional plasmids including the H5N1 NA segment (wild-type or mutant) and the remaining 7 (NP, PA, PB1, PB2, HA, M and NS) segments of A/Québec/144147/09 pH1N1 were co-transfected into MDCK-HEK293T co-cultures using the Lipofectamine 2000 reagent (Invitrogen, Carlsbad, CA) according to the manufacturer’s instructions. Supernatants were collected 72 h post-transfection and used to inoculate MDCK cells.

Viral stocks were produced by inoculating a 75 cm^2^ flask of confluent MDCK cells with MEM supplemented with HEPES and 1 μg/ml with N-tosyl-L-phenylalanine chloromethyl ketone (TPCK)-treated trypsin (Sigma, Oakville, ON, Canada). Neuraminidase and hemagglutinin genes of rescued viruses were sequenced (Sanger) to confirm the absence of undesired mutations.

All experiments with infectious H5N1 viruses were conducted in a certified biosafety level (BSL) 3 laboratory in Quebec City or a BSL4 laboratory in Winnipeg, both in Canada.

### Antivirals

BXA, the active form of baloxavir marboxil (BXM), was synthesized at Shionogi & Co. Ltd, (Osaka, Japan) and diluted in dimethyl sulfoxide (DMSO) to a stock concentration of 10 mM. OSV carboxylate, the active form of OSV phosphate (Hoffmann-La Roche; Basel, Switzerland), zanamivir (ZAN; GlaxoSmithKline, Stevenage, United Kingdom) and PER (BioCryst, Birmingham, USA) were diluted in sterile water to a stock concentration of 10 mM.

### Plaque assays

Viral stocks were titrated using plaque assays in MDCK cells. Briefly, confluent 6-well plates were incubated 1 h at 37°C with 10-fold serial dilutions of each virus. After viral adsorption, the inoculum was removed, then cells were covered with an overlay composed of MEM, 1 µg/ml TPCK-treated trypsin, HEPES and 0.8% agarose then further incubated at 37°C for 3 days. The overlay was removed, cells were stained for 10 min with a 0.8% crystal violet solution and rinsed with water. Viral plaque number was determined on dried plates and plaque size was measured using the free GIMP (GNU Image Manipulation Program) software.

### Assessment of viral replication capacities

To investigate replication capacities of both original BC viral strains and 7:1 reassortants, MDCK cells were inoculated at a multiplicity of infection (MOI) of 0.001 PFU/cell. Confluent 24-well plates were incubated 1 h at 37°C. Then, the inoculum was replaced with a medium solution consisting of MEM, 1 µg/ml TPCK-treated trypsin, HEPES and antibiotics. At 24, 48, 72 and 96 h post-infection (hpi), cell supernatants were harvested and immediately frozen at −80°C until viral titer determination. Each time point was done in triplicate in two independent experiments. Cell supernatants were titrated using TCID_50_ assays and/or RT-qPCR assays as described below.

### TCID_50_ assays

Confluent MDCK cells (10,000 cells/well) in 96-well plates were washed with warm PBS and infected with 10-fold serial dilutions of supernatants in MEM containing TPCK-treated trypsin (1 µg/ml), HEPES and antibiotics. Plates were then incubated at 37 °C with 5% CO_2_ for 6 days. Cytopathic effects were visualized by light microscopy and virus titers, expressed as log_10_ TCID_50_/mL, were calculated as described by Reed and Muench [23].

### Quantitative real-time RT–PCR (RT-qPCR) assays

RNA extraction was performed using the EZ1&2 Virus Mini Kit and the EZ2 connect robot (both from Qiagen) following the manufacturer’s instructions. RT-qPCR assays were performed with the QuantiTect Virus + ROX Vial Kit (Qiagen). Amplification was performed with the LightCycler 480 (Roche) using the following conditions: 50 °C for 20 min, 95 °C for 5 min, followed by 45 cycles of 95 °C for 5 s, 60 °C for 10 s and 72°C for 20 s. Sequences of forward primer, reverse primer and probe were 5’-CATGGARTGGCTAAAGACAAGACC-3’, 5’-AGGGCATTTTGGACAAAMCGTCTA-3’ and 5’-TGCAGTCCTCGCTCACTGGGCACG-3’, respectively. Several dilutions of an appropriate influenza matrix gene plasmid were used to quantify viral RNA yields [24].

### Susceptibility to BXA and OSV by plaque reduction assays

Susceptibility to BXA and OSV was determined following a classical plaque reduction assay. The protocol used is similar to the one described above, except that agarose overlays contained different drug concentrations. BXA and OSV were used at final concentrations ranging from 0.5–8 nM and 5–405 nM, respectively. The 50% inhibitory concentrations (IC_50_) were determined using the GraphPad Prism 10 software.

### Neuraminidase expression and purification

The NA genes of BC-WT and BC-H275Y viruses were amplified by PCR and cloned into pCAGGS expression plasmid. Recombinant plasmids were sequenced and used to transfect FreeStyle 293 F cells using an ExpiFectamine 293 transfection reagent (Invitrogen, Waltham, MA, USA). Transfected cells were left at 37 °C for one week before being pelleted and supernatants were filtered using a 0.22 µm filter. The recombinant NA proteins were purified using nickel-affinity columns and dialyzed against Phosphate-Buffered Saline (PBS) [25]. The purity of the recombinant NA proteins was assessed by staining with Coomassie Blue and loading on SDS-PAGE gels. The NAs were stored in aliquots at −80 °C until further use. Aliquots of 1 μg/mL of purified NAs were used in the NA assays for the determination of 50% inhibitory concentration of IC_50_ and NA activity.

### Neuraminidase inhibition assays and activity

The NAI resistance phenotype to OSV, ZAN and PER was determined by NA inhibition assays using the 2’-(4-methylumbelliferyl)-α-D-N-acetylneuraminic acid (MUNANA, Sigma, St-Louis, MO, USA) substrate [26], with minor modifications. Briefly, the quantities of recombinant neuraminidases used were standardized to NA activity equivalent to 8- to 10-fold that of the background as measured by the production of a fluorescent product from the MUNANA substrate. The drug resistance phenotype was determined by the extent of NA inhibition after incubation with serial three-fold dilutions of the NAI (final concentrations ranging from 0 to 1800 nM). The IC_50_ for each NAI was calculated from the dose–response curve. Neuraminidases were considered to have reduced inhibition (RI) if they showed a 10–100-fold increase in their IC_50_ values when compared to the WT. Neuraminidases were considered to have highly reduced inhibition (HRI) to a drug if their IC_50_ value was increased by >100-fold compared to the WT [27]. A recombinant neuraminidase protein originating from a harbour seal A(H5N1) strain (B1.2 genotype, A/Harbour_Seal/QC/FAV-0836-5/2022) was used as a control.

The NA activity was measured using the MUNANA-based NA assay by quantifying substrate fluorescence with equal quantities of each protein.

### Experimental mouse infections

Groups of six 4- to 6-week-old BALB/c mice (3 male and 3 female) (Charles River, ON, Canada) were infected under isoflurane anaesthesia by intranasal inoculation of serial 10-fold dilutions ranging from 10^1^ to 10^5^ plaque forming units (PFUs) of WT or H275Y mutant A(H5N1) viruses in 50 µl (25 µL per nare) of MEM containing 0.1% bovine serum albumin. Animals were weighed daily for 18 days, monitored for clinical signs and sacrificed if they exceeded pre-approved clinical scores requiring euthanasia (body and coat condition, exceeding 20% weight loss, respiratory rate and/or distress and appearance of signs of central nervous system (CNS) infection including paralysis, ataxia or loss of righting reflex). LD_50_ values were calculated according to the method of Reed and Muench [23].

To evaluate viral replication *in vivo*, additional groups of 6 mice (3 male and 3 female) were infected with 10^4^ PFUs of the WT virus or its H275Y variant and, on day 4 post-inoculation (p.i.), animals were exsanguinated via cardiac bleed and their organs (lung, heart, spleen, kidney, nasal turbinate and brain) were collected and frozen at −80 °C until they were processed. Samples were weighed, placed in MEM, and homogenized in a Bead Ruptor Elite Tissue Homogenizer (Omni) for 30 sec at a speed of 4 m/s. Homogenates were clarified by centrifugation at 1500 x g for 10 min and 10-fold serial dilutions of tissue homogenates were used to infect MDCK cells, in triplicate. TCID_50_ values per gram of tissue were calculated by the same method as above [23].

### Ethical statement

Animal experiments were approved by the Animal Care Committee at the Canadian Science Center for Human and Animal Health per the guidelines provided by the Canadian Council on Animal Care under animal use (document H-22-013). All Infectious work in animals with HPAIV A(H5N1) was performed in the containment level 4 (CL-4) laboratory at the National Microbiology Laboratory (NML) of the Public Health Agency of Canada. Animals were acclimatized 5–7 days before the beginning of all experiments. All animals were monitored and weighed daily throughout the experiments and were provided food and water *ad libitum*.

### Statistics

Statistical analyses were performed on GraphPad Prism 10 software. For plaque size, NA activity and *in vivo* infectious titer comparisons, prior statistical analysis of differences was performed using a Shapiro–Wilk normality test on groups of data. Then, a two-by-two comparison was performed using either an unpaired t test with or without a Welch’s correction (Welch’s t test) if the variance did not assume an equal distribution (Fisher test) or a Mann–Whitney test if the distribution was non-Gaussian. For *in vitro* replication kinetics and weight changes, statistical analyses of differences were performed using a two-way ANOVA test and a mixed-effects model, respectively, both combined to a Šídák's multiple comparisons test. All statistical tests were analyzed with a significance level of 0.05 and were two-sided when relevant.

## Results

### Genomic analysis of H5N1 BC strains

The eight viral segments of both BC influenza A(H5N1) strains were previously amplified by RT–PCR and sequenced [7]. In addition to the substitution at codon 275 of the NA amino acid sequence (H in the WT and Y in the mutant), which is a well-known determinant of resistance to OSV and PER, a few other amino acid changes were identified between the two strains and were screened for phenotypic effects using FluSurver (http://flusurver.bii.a-star.edu.sg) (Table 1).

**Table 1.** Amino acid changes between H5N1-BC strains of interest.

Gene	Location	BC-WT amino acid	BC-H275Y amino acid	Impact
NP	9	S	T	RNP phosphorylation and cellular traffic
PB1	382	D	E	–
PA	168	R	I	–
NA	275	H	Y	High level resistance to OSV and PER.
				Decreased viral fitness in some viral backgrounds.

The NP-S9T mutation may affect viral replication as the phosphorylation of the NP-9S plays a crucial role in the RNP nuclear import [28]. However, no data suggest that the S9T substitution could affect viral replication currently and phosphorylation of threonine can be achieved in eukaryotic cells. The NA of the BC H5N1 isolates shares a relatively high level of amino acid sequence homology (89.6%) with that of a 2009 pandemic H1N1 (pdm09) virus (Figure S1). Noteworthy, we noticed the presence of three amino acid differences (V241I, T289M and N369S) between the H1N1 (pdm09) and H5N1 neuraminidases. The V241I, T289M and N369 K substitutions were previously reported to exert a permissive role in the A(H1N1)pdm09 background restoring the viral fitness impaired by the H275Y NA substitution both *in vitro* and *in vivo* [13,29,30].

**Figure 1.**
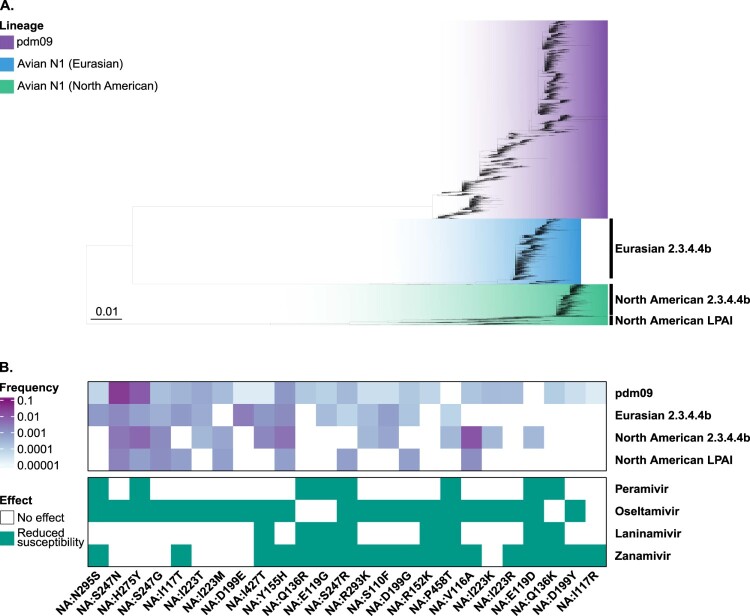
Phylogenetic analysis of avian N1 and A(H1N1)pdm09 lineages and NAI resistance mutations in North America. (A) Maximum-likelihood phylogenetic tree of unique pdm09 and avian lineage N1 neuraminidase genes collected in North America between 2000-01-01 and 2026-03-31 (n = 14,995). Avian lineage N1 genes of Eurasian and North American origin isolated from clade 2.3.4.4b highly pathogenic avian influenza viruses and LPAI viruses are marked on the tree with vertical bars. The scale bar represents tree branch length in substitutions per site (B) Frequency of amino acid substitutions detected among N1 lineages in North America (top) that confer reduced susceptibility to PER, OSV, laninamivir and/or ZAN (bottom) (n = 69,942).

Our phylogenetic analysis confirmed the North American origin of both BC-H5N1 neuraminidase genes (Figure 1A), as already described [7]. Moreover, the frequency of the NA-H275Y substitution was lower in N1 avian lineages (0.062–0.53%) than in the A(H1N1)pdm09 lineage (0.88%) (Figure 1B).

### In vitro susceptibility of H5N1 BC strains to OSV and BXA

**Figure 2.**
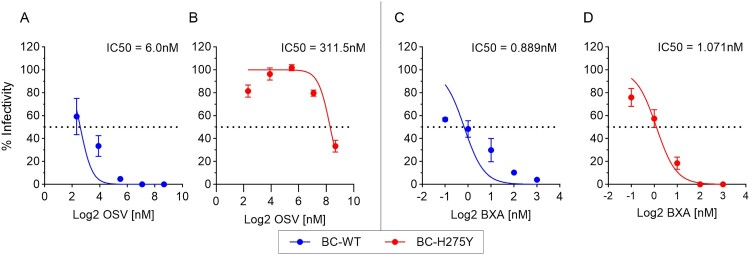
*In vitro* antiviral activity of oseltamivir (OSV) and baloxavir acid (BXA) against H5N1 BC strains. Susceptibility of BC-WT strain to OSV (A) and BXA (C). Susceptibility of BC-H275Y strain to OSV (B) and BXA (D). Susceptibilities were assessed using plaque reduction assays on MDCK cells. Compound concentrations are presented in log_2_ scale. Dose response curves were generated using a non-linear regression analysis (GraphPad Prism 10 software).

We first determined the susceptibility profile of both BC-H5N1 strains to OSV and BXA in plaque reduction assays using MDCK cells (Figure 2). OSV and BXA inhibited viral replication of the BC-WT strain with IC_50_ values of 6.00 and 0.89 nM, respectively. As expected, BC-H275Y demonstrated a reduced inhibition profile (IC_50_ = 311.5 nM), representing a 51.92-fold increase compared to the BC-WT strain whereas it remained susceptible to BXA (IC_50_ = 1.071 nM).

### Enzymatic activity and susceptibility to NAIs of recombinant NA proteins

**Figure 3.**
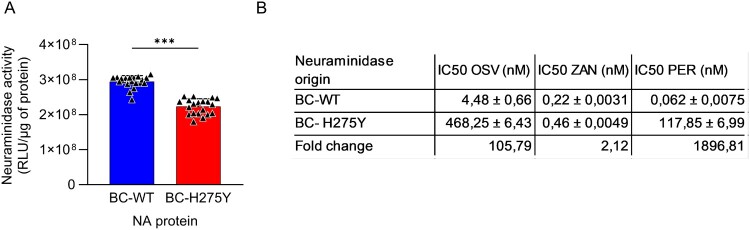
NA activity and susceptibility to NA inhibitors of recombinant BC-H5N1 NA proteins. (A) NA activity of recombinant BC-WT and BC-H275Y neuraminidases. ***p < 0.001 (Mann-Whitney test). Data represent mean results ± SD from two independent experiments performed in 7–13 replicates. (B) IC_50_ values of both recombinant BC-H5N1 NAs. Data represents mean results ± SD from two independent experiments performed in duplicates.

By using recombinant NA proteins, we further characterized enzymatic activities (Figure 3A) and susceptibility profiles to the common NA inhibitors OSV, ZAN and PER for both BC-H5N1 purified NA proteins (Figure 3B).

Recombinant BC-WT and BC-H275Y NAs showed activities of 2.94 ± 0.17 × 10^8^ and 2.23 ± 0.22 × 10^8^ relative fluorescence units (RFU) per microgram of protein, respectively. This difference highlighted a decrease of 24.15% in NA activity induced by the H275Y substitution (*p* < 0.001) (Figure 3A).

In NA inhibition assays, OSV, ZAN and PER showed a strong inhibitory effect against the BC-WT NA with IC_50_ values of 4.48, 0.22 and 0.062 nM, respectively. By contrast, the BC-H275Y NA exhibited a highly reduced inhibition phenotype against OSV and PER with IC_50_ values of 468.25 nM (1896.81-fold increase vs the WT NA) and 117.85 nM (105.79-fold increase vs the WT NA) but it remained susceptible to ZAN with an IC_50_ value of 0.46 nM (2.12-fold increase compared to the WT NA protein) (Figure 3B). As a comparison, IC_50_ values for a Harbour seal H5N1 NA (genotype B1.2) were 5.56, 0.19 and 0.07 nM for OSV, ZAN and PER, respectively.

### Viral plaque size and in vitro replication kinetics

**Figure 4.**
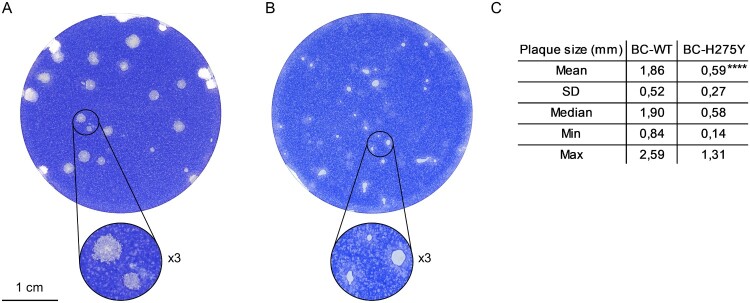
Plaque size of BC-WT and H275Y viruses in MDCK cells. Viral plaque on MDCK cells in six-well plates for BC-WT (A) and BC-H275Y (B) at 3 days p.i. (C) Plaque size characteristics. Plaque sizes were determined using 17 and 30 representative plaques for BC-WT and H275Y viruses, respectively (C). ****p < 0.0001 (Welch’s t test).

We further evaluated viral fitness of BC-H5N1 strains *in vitro* (Figure 4A and B). Viral plaques produced by the BC-WT strain were significantly larger than those produced by the BC-H275Y mutant virus on MDCK cells with mean plaque sizes of 1.86 ± 0.52 mm and 0.59 ± 0.27 mm, respectively (*p* < 0.0001) (Figure 4C).

**Figure 5.**
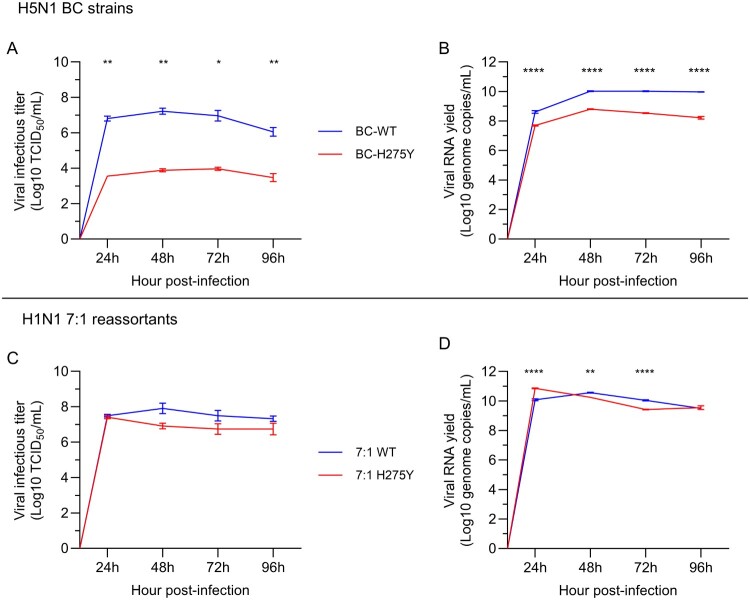
*In vitro* replication kinetics of native BC-H5N1 strains and 7:1 reassortants. Viral replication kinetics were performed on MDCK cells over 4 days. Data represent means ± SD from triplicates. Viral infectious titers (A and C) and viral RNA yields (B and D) were determined for H5N1 BC strains and 7:1 reassortants. *p < 0.05; **p < 0.01 and ****p < 0.0001 (Two-way ANOVA test). Similar results were observed in two independent replicates.

In replication kinetics experiments, the BC-WT virus exhibited significantly higher titers compared to the BC-H275Y mutant for all 4 time points with viral titers ranging from 6.05 to 7.22 log_10_ TCID_50_/mL compared to 3.47 to 3.96 log_10_ TCID_50_/mL, respectively (Figure 5A). A similar pattern was observed when assessing viral RNA copy numbers that ranged from 8.61 to 10.02 log_10_ copies/mL for the WT and from 7.70 to 8.53 log_10_ copies/mL for the mutant (Figure 5B).

By contrast, the mutant H275Y 7:1 reassortant (7:1 H275Y) was found to grow at an equivalent level compared to the WT 7:1 reassortant (7:1 WT) when assessed by TCID_50_, with viral titers ranging from 7.32 to 7.91 log_10_ TCID_50_/mL and to 6.74 to 7.41 log_10_ TCID_50_/mL for the 7:1 WT and 7:1 H275Y reassortants, respectively (Figure 5C). A similar pattern was observed when assessing viral RNA copy numbers that ranged from 9.51 to 10.57 log_10_ copies/mL for the 7:1 WT reassortant and from 9.43 to 10.86 log_10_ copies/mL for the 7:1 H275Y reassortant. Viral RNA copies were even higher for the 7:1 H275Y reassortant at 24 h p.i. Significant statistical differences were measured at each time point of the assay and can be attributed to the temporal variation in replication peaks. Indeed, the mutant reassortant reached its replication peak at 24 h, whereas the WT reassortant reached its peak at 48 h (Figure 5D).

### Virulence of BC-WT and BC-H275Y H5N1 strains in mice

**Figure 6.**
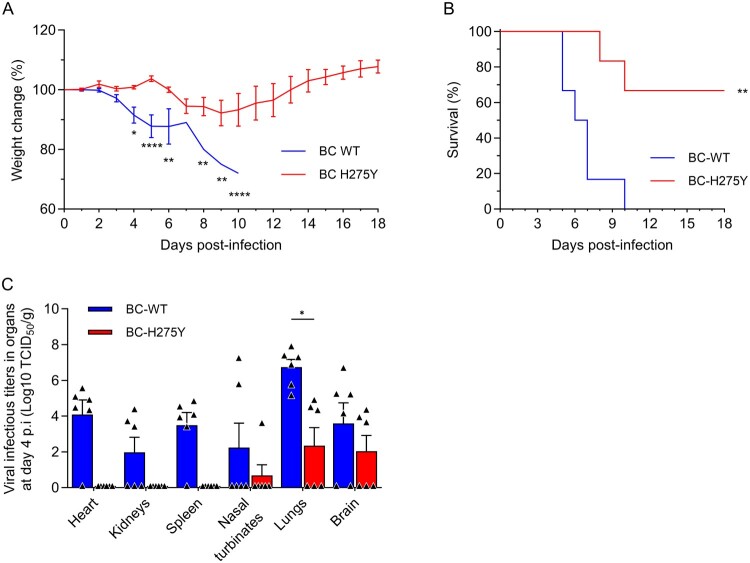
*In vivo* characterization of H5N1 BC strains. Groups of 6 BALB/c mice (n = 6, 3 males and 3 females) were infected with 10^4^ PFUs of BC-WT or BC-H275Y strains. (A) Body weight changes of infected mice. Mean percent weight changes ± SEM (as compared to initial weights) of mice inoculated intranasally with BC-WT or BC-H275Y strain were recorded daily until day 18 post-infection. *, *p* < 0.05; **p < 0.01 and ****p < 0.0001 for comparison between the two groups (Mixed-effects model). (B) Survival curves of groups of mice inoculated intranasally with 10^4^ PFU of BC-WT or BC-H275Y strains. **, *p* = 0.0038 for comparison between the two groups (Mantel-Cox test). (C) Viral titers in organ samples of mice inoculated intranasally with BC-WT or BC-H275Y strains. Animals were sacrificed at day 4 p.i. Data represent means ± SD. *, *p* = 0.013 for comparison between the two groups (Mann-Whitney test).

Experimental infection of BALB/c mice with the WT and H275Y H5N1 BC viruses resulted in apparent signs of infection, including weight loss that was more prominent between days 4 and 10 p.i. in the WT group (Figure 6A). The LD_50_ values evaluated for the two viruses at the acute phase of infection were 1.78 × 10^3^ PFUs and 8.71 × 10^4^ PFUs for the WT and the H275Y variant, respectively (Figure 6B and Figure S2).

Positive viral cultures were obtained using nasal turbinates (2/6 vs 1/6), lung (6/6 vs 3/6) and brain (4/6 vs 3/6) samples from the WT and H275Y groups of animals, respectively, at day 4 p.i. after infection with 10^4^ PFUs. Mean lung viral titers were significantly higher in the WT group compared to the mutant [6.74 Log10(TCID50/g) vs 2.34 Log10(TCID50/g); *p*<0.05] (Figure 6C). Brain and nasal titers were also higher in the WT group but with no statistical significance. On the other hand, only the WT group had positive viral cultures for heart (5/6), kidney (3/6) and spleen (5/6) samples.

## Discussion

Previous H5N1 oseltamivir resistance assessments focused on early Goose/Guangdong clade 1 poultry isolates, reporting moderate H275Y resistance by NA inhibition [31]. The purpose of the present study was to compare *in vitro* and *in vivo* properties of WT and H275Y HPAIV H5N1 viruses belonging to clade 2.3.4.4b, genotype D1.1. Here we report that the H275Y substitution confers highly reduced susceptibility to OSV and PER and is associated with impaired viral replicative capacity *in vitro* and reduced virulence in experimentally-infected mice when compared to the WT counterpart.

HPAI H5N1 infections can be associated with severe infections in humans requiring efficient therapeutic options. The influenza NAIs, including oral OSV (the most widely prescribed NAI compound), ZAN, PER and laninamivir have constituted the main class of anti-influenza agents over the last two decades [10]. BXM, whose active compound (BXA) targets the cap-dependent endonuclease activity of the influenza polymerase acidic (PA) protein, is the first RNA polymerase inhibitor [32] approved for uncomplicated influenza A and B infections. Based on observational studies, the Centers for Disease Control (CDC) and the World Health Organization (WHO) have recommended OSV as the primary medication for the treatment of HPAI H5N1 infections as well as for post-exposure prophylaxis [33]. However, as for other antivirals, the use of OSV may lead to the emergence of drug-resistant variants. The most frequent NA change conferring resistance to OSV is the H275Y NA substitution, which was described in influenza viruses of the N1 subtype, including seasonal and pandemic A(H1N1) viruses. This mutation was also identified in HPAI H5N1 variants isolated from patients who received OSV treatment [34] as well as those who received prophylactic doses of OSV [35]. The H275Y substitution was also induced in influenza A(H5N1) strains from different clades after *in vitro* passages under OSV pressure [36]. However, this mutation has not been detected in 8 poultry workers with cluster 2.3.4.4b [37].

Determination of drug phenotype for BC-WT and BC-H275Y recombinant NAs as well as 7:1 reassortants by NA inhibition assays showed expected results with the H275Y substitution being associated with highly reduced susceptibility to OSV and PER (more than 100-fold increase in IC_50_ values compared to the WT) while the two viruses remained susceptible to ZAN. We also obtained a susceptible phenotype to BXA for both BC-WT and BC-H275Y viruses by plaque reduction assay with IC_50_ values of ≈ 1 nM, in line with those observed for bovine H5N1 strains [38]. The clinical effectiveness of BXM for treatment of HPAI H5N1 infections is not well documented; nevertheless, recommendations on its use could be extrapolated from *in vitro* and *in vivo* studies involving HPAI H5N1 strains as well as on the available clinical experience with seasonal influenza virus infection. While our *in vitro* assays provide a robust assessment of NAI susceptibility for the BC H5N1 isolates, it will be interesting to confirm these results in an appropriate animal model. Overall, these results suggest the potential use of ZAN and BXM as alternative therapy against HPAI H5N1 infections involving H275Y variants.

The acquisition of mutations conferring antiviral drug resistance is often associated with a fitness cost that may vary depending on the viral background. We previously showed that the H275Y substitution exerted a more potent impact on the fitness of old influenza A(H1N1) viruses (such as A/WSN/1933 and A/Mississippi/3/2001) than that of more recent A/Brisbane/59/2007-like viruses [9]. Indeed, most influenza A/Brisbane/59/2007-like strains that circulated worldwide during the 2008–2009 season were H275Y variants [39] suggesting conserved viral fitness and transmissibility of H275Y in that viral background. The possible molecular explanation for this observation was provided by Bloom and colleagues who showed that V234M and R222Q secondary NA substitutions, present in A/Brisbane/59/2007-like viruses, may have counteracted the compromising impact of the H275Y substitution [14]. Our group further demonstrated the compensatory nature of these mutations in recombinant viruses by reverting the latter two substitutions (i.e. M234 V and Q222R) in NA of the OSV-resistant clinical A/Brisbane/59/2007 H275Y variant [12]. A set of potential so-called permissive NA substitutions, including V241I, T289M and N369 K, was also proposed to restore the fitness of influenza A(H1N1)pdm09 H275Y viruses [13,29].

Regarding the fitness impact of the H275Y substitution in the 2.3.4.4b clade, our experiments showed a reduced replication *in vitro* for the mutant compared to the WT HPAI H5N1 counterpart, as revealed by smaller viral plaque size (∼50% diameter reduction) and lower viral yields in multi-cycle replication kinetics experiments. The BC-H275Y mutant was also less virulent in experimentally-infected mice although it induced rapid lethality of animals when the highest doses of the virus were given. The reduced fitness demonstrated for BC-H275Y compared to BC-WT depends on reduced NA activity observed in NA assays using recombinant BC-H5N1 NA (Figure 3A), likely disrupting the HA–NA functional balance. Such defect can potentially be restored by permissive NA substitutions. When comparing the NA sequences of BC-H5N1 viruses with those of seasonal viruses of the H1N1 subtype, we found that BC-H5N1 NAs were more related to those of A(H1N1)pdm09 viruses. Interestingly, we noted the presence of the permissive NA mutations V241I and T289M previously characterized in the A(H1N1)pdm09 background in addition to the N369S change (instead of N369 K) (Figure S1). However, these substitutions did not restore the fitness of the H275Y mutant in the 2.3.4.4b H5N1 background. Since the nature of NA substitutions with a permissive role was shown to vary depending on the viral background, it was possible that the V241I, T289M and N369S would be functional in A(H1N1)pdm09 viruses only. To address this question, we rescued 7:1 reassortants where the BC-H5N1 WT or H275Y NA segment was mixed with the HA and the remaining 6 segments (PA, PB1, PB2, NP, M and NS) of the A(H1N1)pdm09 virus. We demonstrated the absence of a fitness defect for the reassortant virus containing the BC-H5N1 H275Y NA segment as compared to its WT counterpart *in vitro* (Figure 5). These results suggest that permissive mutations efficiently restored HA–NA functional balance in an A(H1N1)pdm09 background. Enhanced interactions between the NA and the internal gene constellation may also have played a role.

As the impact of epidemic and pandemic influenza infections is influenced by the efficiency of influenza transmission between humans, it would be of great interest to assess the ability of the H275Y H5N1 variant to be transmitted in a suitable mammalian model, such as ferrets, looking at both droplet and aerosol transmission.

It has been well described that the H275Y NA substitution can be selected under OSV pressure either clinically or in experimental *in vitro/in vivo* procedures. However, to the best of our knowledge, there was no use of OSV in poultry that could explain the emergence of the H275Y NA substitution in BC farms. One possible explanation could be poultry exposure to antivirals in the environment (e.g. via contaminated water near human treatment centers) [40]. Another possibility is cross-species contamination where the mutation would emerge in humans and spills back into poultry but the only human Canadian case with H5N1 infection so far was not infected by this mutant [6]. A more probable scenario seems to be that the H275Y substitution has emerged spontaneously as influenza viral replication involves an error-prone RNA-dependent RNA polymerase, with random mutations occurring frequently. Such mutation could potentially favour a more optimal balance between HA and NA in some viruses.

OSV is considered the preferred antiviral for the control of a potential influenza pandemic involving avian influenza viruses such as H5N1. Consequently, stockpiles of this compound have been established in several countries worldwide. This study and other observations suggest that it would be unwise not to consider alternatives in the event of the emergence of the H275Y substitution. This should include the consideration of other antivirals such as BXM, as well as combinations of antivirals with different mechanisms of action, i.e. ZAN (NAI) and BXM (polymerase inhibitor). Also, our findings confirm the need for extensive surveillance studies for influenza drug resistance not restricted to humans.

## Supplementary Material

Revised supplementary figures H5N1.docx
