# Supplementary material for: Characterization of oseltamivir-resistant A(H5N1) clade 2.3.4.4b, genotype D1.1 variants identified in poultry farms of British Columbia, Canada
Source: Emerg Microbes Infect. 2026 Jul 8;15(1):2686474. doi: 10.1080/22221751.2026.2686474 (PMC13353464; doi:10.1080/22221751.2026.2686474)
Supplement: Revised supplementary figures H5N1.docx [file TEMI_A_2686474_SM9575.docx]

Supplementary figures

BC-WTH5N1 1 MNPNQKIITIGSICMVIGIISLVLQIGNIISIWVSHSIQTGNQNHPETCNQSVITYENNT

Cal-pDM09 1 MNPNQKIITIGSVCMTIGMANLILQIGNIISIWISHSIQLGNQNQIETCNQSVITYENNT

************ ** ** * ********** ***** **** **************

BC-WTH5N1 61 WVNQTYINISNTNLIAEQAVDPVALAGNSSLCPISGWAIYSKDNGIRIGSKGDVFVIREP

Cal-pDM09 61 WVNQTYVNISNTNFAAGQSVVSVKLAGNSSLCPVSGWAIYSKDNSVRIGSKGDVFVIREP

****** ****** * * * * ********* ********** **************

BC-WTH5N1 121 FISCSHLECRTFFLTQGALLNDKHSNGTVKDRSPYRTLMSCPVGEAPSPYNSRFESVAWS

Cal-pDM09 121 FISCSPLECRTFFLTQGALLNDKHSNGTIKDRSPYRTLMSCPIGEVPSPYNSRFESVAWS

***** ********************** ************* ** **************

BC-WTH5N1 181 ASACHDGISWLTIGISGPDNGAVAVLKYNGIITDTIKSWRSNILRTQESECACINGSCFT

Cal-pDM09 181 ASACHDGINWLTIGISGPDNGAVAVLKYNGIITDTIKSWRNNILRTQESECACVNGSCFT

******** ******************************* ************ ******

BC-WTH5N1 241 IMTDGPSNGQASYKIFRIEKGKVVKSVELNASNYHYEECSCYPDASEVMCVCRDNWHGSN

Cal-pDM09 241 VMTDGPSNGQASYKIFRIEKGKIVKSVEMNAPNYHYEECSCYPDSSEITCVCRDNWHGSN

********************* ***** ** ************ ** ***********

BC-WTH5N1 301 RPWVSFNQNLEYQIGYICSGVFGDNPRPSDGTGSCGPVSSNGAYGVKGFSFKYGNGVWIG

Cal-pDM09 301 RPWVSFNQNLEYQIGYICSGIFGDNPRPNDKTGSCGPVSSNGANGVKGFSFKYGNGVWIG

******************** ******* * ************ ****************

BC-WTH5N1 361 RTKSTSSRSGFEMIWDPNGWTETDSSFSVKQDIVAITDWSGYSGSFVQHPELTGLDCMRP

Cal-pDM09 361 RTKSISSRNGFEMIWDPNGWTGTDNNFSIKQDIVGINEWSGYSGSFVQHPELTGLDCIRP

**** *** ************ ** ** ***** * ******************* **

BC-WTH5N1 421 CFWVELIRGRPKENTIWTSGSSISFCGVNSDTVGWSWPDGAELPFTIDK

Cal-pDM09 421 CFWVELIRGRPKENTIWTSGSSISFCGVNSDTVGWSWPDGAELPFTIDK

*************************************************

Figure S1: Alignment of the whole amino acid sequences of the British Columbia A(H5N1) and the California A(H1N1)pdm09 wild-type neuraminidases. Sequence analysis was performed using the CLUSTAL program. Potential permissive substitutions are highlighted in yellow (13, 29, 30).

Figure S2: Mouse weight changes and survival following H5N1 BC strains infection. Groups of 6 BALB/c mice (n = 6, 3 males and 3 females) were infected with serial dilutions, from 10^5^ to 10^1^ PFUs of BC-WT or BC-H275Y strains. Body weight changes of BC-WT (A) and BC-H275Y (C) infected mice. Percent survival of BC-WT (B) and BC-H275Y (D) infected mice.
